# Supplementary material for: Inhibition of Amyloid-β (Aβ)-Induced Cognitive Impairment and Neuroinflammation in CHI3L1 Knockout Mice through Downregulation of ERK-PTX3 Pathway
Source: Int J Mol Sci. 2024 May 19;25(10):5550. doi: 10.3390/ijms25105550 (PMC11122210; doi:10.3390/ijms25105550)
Supplement: Supplementary file 1 [file ijms-25-05550-s001.zip › ijms-2942258-supplementary.pdf]

Supplementary information for

Inhibition of amyloid-β (Aβ)-induced cognitive impairment and neuroinflammation in CHI3L1 knockout mice through downregulation of ERK-PTX3 pathway

Hyeon Joo Ham[1], Yong Sun Lee[1], Ja Keun Koo[1], Jaesuk Yun[1], Dong Ju Son[1], Sang-Bae Han[1], and Jin Tae Hong[1]

[1]College of Pharmacy and Medical Research Center, Chungbuk National University, Osongsaengmyeong 1-ro, Osong-eup, Heungdeok-gu, Cheongju, Chungbuk, 28160, Republic of Korea

*Correspondence: Dr. Jin Tae Hong (jinthong@chungbuk.ac.kr), College of Pharmacy and Medical Research Center, Chungbuk National University, Osongsaengmyeong 1-ro, Osong-eup, Heungdeok-gu, Cheongju, Chungbuk, 28160, Republic of Korea, Tel: +82-043-261-2813, Fax: +82-043-268-2732.

Supplementary Table S1. List and sequences of qPCR primers for mRNA expression

	Forward (5’→3’)	Reverse (5’→3’)	Species
β-actin	GGCTGTATTCCCCTCCATCG	CCAGTTGGTAACAATGCCATGT	Mouse
Tnf	TCTTCTCATTCCTGCTTGTGG	CACTTGGTGGTTTGCTACGA	Mouse
Il1b	CCTTCCAGGATGAGGACATGA	TGAGTCACAGAGGATGGGCTC	Mouse
Il6	GAGGATACCACTCCCAACAGACC	AAGTGCATCATCGTTGTTCATACA	Mouse
Ccl2	TTAAAAACCTGGATCGGAACCAA	GCATTAGCTTCAGATTTACGGGT	Mouse
Cd16	TTTGGACACCCAGATGTTTCAG	GTCTTCCTTGAGCACCTGGATC	Mouse
Cd32	AATCCTGCCGTTCCTACTGATC	GTGTCACCGTGTCTTCCTTGAG	Mouse
Cd68	TTCTGCTGTGGAAATGCAAG	AGAGGGGCTGGTAGGTTGAT	Mouse
Cd86	TGTTTCCGTGGAGACGCAAG	TTGAGCCTTTGTAAATGGGCA	Mouse

Supplementary figure S1. Effect of CHI3L1 deficiency on Aβ deposition

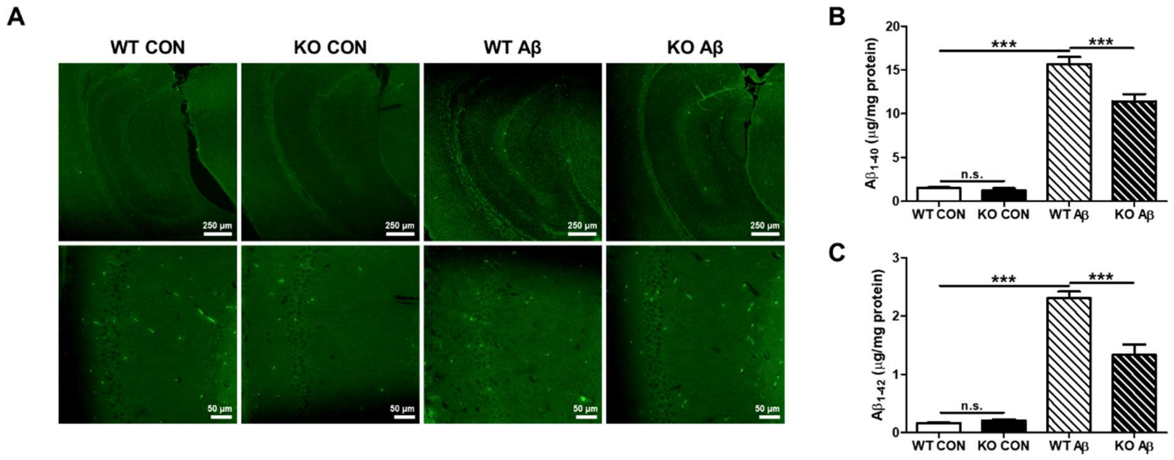

(A) The deposition of Aβ plaques in hippocampus was determined by Thioflavin S staining. (B) The levels of $A\beta_{1\text{—}42}$ and (C) $A\beta_{1\text{—}40}$ in mice hippocampus as 100 μg of tissue lysate, were assessed using the specific ELISA kits. Each value is mean ± S.E.M. from 8-10 samples. ***, Significantly different between the two groups ($p < 0.001$).

Supplementary figure S2. Relative intensity of each band in Western blots in Figure 3D, 3E, and 6E

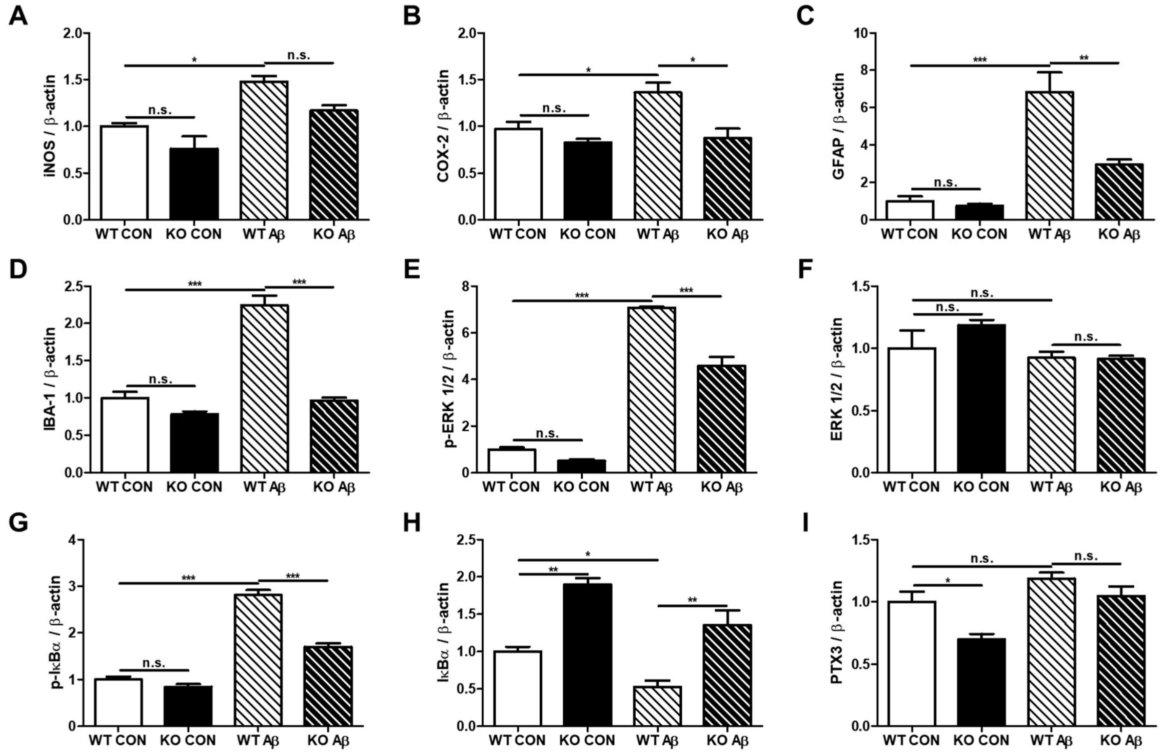

The intensity of each band was measured and the ratio of each protein relative to β-actin was calculated. (A, B, C, D) The relative ratio of each protein in Figure 3D were calculated. (E, F, G, H) The relative ratio of each protein in Figure 3E were calculated. (I) The relative ratio of PTX3 in Figure 6E were calculated. Each value is mean ± S.E.M. from 3 samples. *, Significantly different be-tween the two groups ($p < 0.05$); **, Significantly different between the two groups ($p < 0.01$); ***, Significantly different between the two groups ($p < 0.001$).
